# The evolution of plasticity of dauer larva developmental arrest in the nematode *Caenorhabditis elegans*

**DOI:** 10.1002/ece3.1436

**Published:** 2015-03-02

**Authors:** S Anaid Diaz, Mark Viney

**Affiliations:** School of Biological Sciences, University of BristolTyndall Avenue, Bristol, BS8 1TQ, UK

**Keywords:** *C. elegans*, dauer larvae, evolution, phenotypic plasticity, selection experiment

## Abstract

Organisms can end up in unfavourable conditions and to survive this they have evolved various strategies. Some organisms, including nematodes, survive unfavourable conditions by undergoing developmental arrest. The model nematode *Caenorhabditis elegans* has a developmental choice between two larval forms, and it chooses to develop into the arrested dauer larva form in unfavourable conditions (specifically, a lack of food and high population density, indicated by the concentration of a pheromone). Wild *C. elegans* isolates vary extensively in their dauer larva arrest phenotypes, and this prompts the question of what selective pressures maintain such phenotypic diversity? To investigate this we grew *C. elegans* in four different environments, consisting of different combinations of cues that can induce dauer larva development: two combinations of food concentration (high and low) in the presence or absence of a dauer larva-inducing pheromone. Five generations of artificial selection of dauer larvae resulted in an overall increase in dauer larva formation in most selection regimes. The presence of pheromone in the environment selected for twice the number of dauer larvae, compared with environments not containing pheromone. Further, only a high food concentration environment containing pheromone increased the plasticity of dauer larva formation. These evolutionary responses also affected the timing of the worms’ reproduction. Overall, these results give an insight into the environments that can select for different plasticities of *C. elegans* dauer larva arrest phenotypes, suggesting that different combinations of environmental cues can select for the diversity of phenotypically plastic responses seen in *C. elegans*.

## Introduction

**Figure 1:**
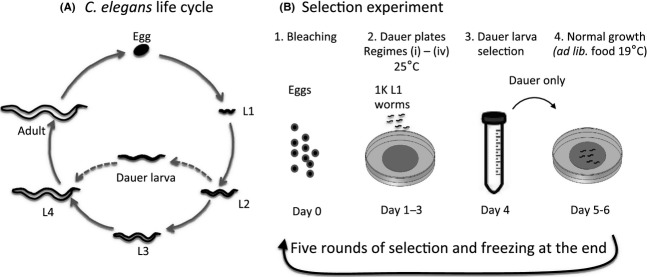
Schematic representation of (A) The *Caenorhabditis elegans* life cycle in which L2s can develop into the L3 stage (solid arrow) or into the dauer larva stage (dotted arrow) when conditions become unfavourable, and (B) The selection protocol.

Organisms have different ways to cope with unfavourable conditions. Some species can developmentally arrest (also known as diapause) to avoid these otherwise unfavourable conditions. Developmental arrest is a well-documented example of developmental phenotypic plasticity among invertebrates and is common among insects and nematodes, as well as other invertebrates (Danks 1987; Denlinger 2002; West-Eberhard 2003; Chen and Glazer 2004). Despite developmental arrest being common in nature, there is no general understanding of the selective pressures that favour this, nor that maintain it in a population. Many free-living nematodes have an alternative, arrested third larval stage form called the dauer larva. Specifically, young larvae have a developmental choice between growing into a “normal” non-dauer larva or arresting their development as a dauer larva. This is best studied in the model nematode *Caenorhabditis elegans* where dauer larvae develop when there is overcrowding and a lack of food (Fig.1A). *Caenorhabditis elegans* dauer larvae are a morphologically distinct, stress resistant, and long lived form and the stage most commonly found in the wild (Félix and Braendle 2010), thus suggesting that this stage is of critical importance in the ecology of *C. elegans*.

There has been extensive study of the molecular and genetic basis of the formation of *C. elegans* dauer larvae (Riddle et al. 1981; Riddle and Albert 1997; Hu 2007). Despite this, there is rather little understanding of how environmental cues contribute to and maintain the dauer larva formation response in nature. In the wild *C. elegans* lives in ephemeral environments, particularly rotting vegetation, and population sizes vary enormously, presumably as food availability waxes and wanes (Félix and Braendle 2010; Petersen et al. 2014). Two major cues for the developmental choice between forming dauer or non-dauer larvae are the concentration of food and of ascaroside-based pheromone molecules in the environment (Ludewig and Schroeder 2014). All worms release the ascarosides and thus the environmental ascaroside concentration is potentially a measure of conspecific population density (Golden and Riddle 1984). Together, the balance between these two cues (food and ascaroside) is thought to be an indication of the likely future conditions that worms will encounter, so that young larvae can decide to develop and reproduce, or to arrest their development as dauer larvae.

We have recently discovered significant variation in the dauer larva formation phenotypes among wild-derived isolates of *C. elegans* when exposed to the same food and ascaroside environments (Diaz et al. 2014). The dauer larva formation phenotype, when assayed in two environments, can be quantified in two ways: (1) the trait's average value across the two environments and (2) the difference in the trait values between the two environments, thus the trait's plasticity, which can also be thought of as its sensitivity to the change in the environment (Viney and Diaz 2012). Thus, *C. elegans* could have a high or low average dauer larva formation and, separately, a high or low plasticity of dauer larva formation (Viney and Diaz 2012). The discovery of this diversity of plasticity of dauer larva arrest phenotypes raises the question of what environmental cues maintain such diversity of phenotypic plasticity. The isolates used in this survey were isolated from different locations, and this could have given rise to different selective pressures, resulting in the different phenotypes observed (Diaz et al. 2014).

Here we investigated how selection in different cue environments affects the dauer larva formation phenotype of *C. elegans*. Specifically, we wished to determine how the plasticity of dauer larva development evolved in response to different food and pheromone environments. To do this, we grew a genetically diverse population in each of four regimes, consisting of combinations of different food environments (high or low concentration) and the presence or absence of a dauer larva-inducing ascaroside molecule and then selected the dauer larvae that formed in each. These four environmental regimes are therefore one or two cue environments.

We found that most of the selection regimes increased the average formation of dauer larvae, compared with the control, unselected population. However, only one selection environment (high food and ascaroside) increased the plasticity of dauer larva formation. Our results give insights into how food and pheromone cue environments interact during selection to evolve plasticity of *C. elegans*’ developmental choice to form dauer larvae. These processes are potentially operating in the natural environment to generate the diversity of phenotypically plastic responses seen in *C. elegans*.

## Methods

To investigate the evolution of the plasticity of formation of *C. elegans* dauer larvae we grew a genetically diverse *C. elegans* population (Teotonio et al. 2012) in four different regimes that differed in the availability of food and in the presence of a synthetic ascaroside and then selected the dauer larvae that developed in these environments. After five rounds of selection we tested the worms in different food environments to measure their plasticity of dauer larva formation.

### *Caenorhabditis elegans* populations and lines

The *C. elegans* population that we used was formed from 16 wild-type isolates using a funnel-cross mating strategy (Teotonio et al. 2012). This population is androdioecious and had previously been maintained for over 100 nonoverlapping generations at a large (10,000) population size and contains high genetic diversity. This population is referred to as G140.A and was kindly made available by H. Teotonio. Unless otherwise stated all *C. elegans* populations were maintained on 90-mm-diameter plates containing 15 mL of NGM agar seeded with 1 mL of *Escherichia coli* OP50 (*ca*. 10^7^–10^8^ live cells per mL).

### Dauer larva selection

We grew the G140.A population in four different selection regimes (Fig.1B), for five generations each, with 10 replicate plates for each selection regime. The four selection regimes were (i) low (2% w/v) food concentration; (ii) high (5%) food concentration; (iii) low food + ascaroside 2 (ascr#2); and (iv) high food + ascr#2. As a control we maintained the G140.A population in unlimited food conditions, without selection, for the same number of generations.

For each generation of selection at the beginning (Day 0) we synchronized the population by bleaching (Teotonio et al. 2012), which is only survived by eggs. These eggs were then maintained without food for 24 h at 19°C during which time the eggs hatched to release first stage larvae (L1s). We determined the number of L1s present 24 h after each bleaching by collecting the larvae in 1 mL of M9. The number of L1s was then determined by counting the number of larvae in each of ten 10 μL samples; thus, we counted ten percent of the sample. For each generation, we then (Day 1) randomly seeded *c*. 1000 L1s on to each of 10 plates of regimes (i)–(iv). These plates were then maintained for 72 h (days 1–3) at 25°C on 90-mm-diameter plates containing 24 mL of dauer agar. The dauer agar consists of 3.3% w/v agar in 51.3 mmol/L NaCl, 1 mmol/L CaCl_2_, 1 mmol/L MgSO_4_, which was autoclaved and then supplemented with cholesterol, phosphate buffer (pH 6.0, Hope 1999) and streptomycin at final concentrations of 0.005 mg/mL, 96 *μ*mol/L, and 0.05 mg/mL, respectively. For ascr#2-containing plates the ascr#2 concentration was 20 mmol/L; the ascr#2 was synthesized as described by Diaz et al. (2014). Plates were seeded with 1 mL of 2% w/v or 5% w/v food consisting of *E. coli* OP50 strain. For each generation of selection (above) or of phenotyping (below), bacteria were grown overnight in LB medium, centrifuged, and prepared by diluting the bacteria in S buffer, as described by Diaz et al. (2014).

On Day 4 dauer larvae were selected. This was performed using sodium dodecyl sulfate (SDS) selection and sucrose density gradient separation (see below). Dauer larvae thus selected were then transferred to NGM plates seeded with *E. coli* OP50 and maintained for 72 h (days 4–6) at 19°C, during which time dauer larvae resumed development and grew into reproductive adults. On Day 6 we determined the number of adult worms that resulted after each round of selection per plate (which is thus a measure of the proportion of dauer larvae selected on Day 4 of the 1000 L1s added on Day 1, for each selection regime and at each generation). The offspring from the adult worms on all the plates were pooled and these were then the starting material for the next round of selection starting at Day 0 (Fig.1B).

After five generations of selection from the Day 6 step we cryopreserved all viable L1s resulting from the 10 replicate plates of each regime. Later, portions of these populations were thawed onto NGM plates seeded with an excess of *E. coli* food. After 48 h we randomly selected five adult hermaphrodites each of which was the founder of an isogenic line that was inbred by single worm self-fertilization for five generations as described by Diaz et al. (2014). The resulting five isogenic lines for each selection regime were then cryopreserved and thawed when required for phenotyping.

### SDS treatment and sucrose density gradient separation

We used an SDS-based selection of dauer larvae, a modified version of that described by Mayer and Sommer (2011). On Day 4 (above), worms were washed from plates with M9 buffer and resuspended in 1% w/v SDS in M9 with gentle agitation for 15 min at room temperature, after which the worms were sedimented by centrifugation at 1650 *g* for 5 min, resuspended in fresh M9, and this repeated three times. After the final wash, sedimented larvae were resuspended in M9 to which an equal volume of 60% w/w sucrose was added. The samples were inverted twice, centrifuged at 50 *g* for 1 min and then, immediately, at 1150 *g* for 3 min. Following this, live worms were in the upper layers, while debris sedimented. The live worm-containing layer was removed and diluted with M9, and the worms sedimented by centrifugation at 1650 *g* for 5 min; this was repeated three times. The final sedimented worms were then transferred to NGM plates (days 4–6, above).

### Assaying dauer larva formation and plasticity

We determined the dauer larva formation phenotypes of (1) each of the five isogenic lines resulting from each of the four ((i)–(iv)) selection regimes and (2) five isogenic lines from the control, unselected population, a total of 25 isogenic lines. These assays were carried out as previously described (Golden and Riddle 1984; Viney et al. 2003; Diaz et al. 2014). Briefly, 30-mm-diameter plates containing 2 mL of dauer larva agar (see “*Dauer larva selection*” section, above) without ascaroside were inoculated with 20 *μ*L of 2% or 5% w/v *E. coli* OP50 food. On each plate, five hermaphrodites of the same age were added and allowed to lay eggs for 3–4 h or until approximately 50 eggs were present on each plate, after which the hermaphrodites were removed. These plates were then maintained at 25°C and after 48 h the dauer and nondauer larvae were counted. Each combination of isogenic line and food concentration was replicated three times. Plasticity of dauer larva formation was defined as the absolute difference in the proportion of dauer larvae that were formed in the 2% and 5% food concentration environments.

### Fecundity and survival

We investigated the lifetime fecundity and the survival of the selected and control lines in ad libitum food conditions. The lifetime fecundity assays were carried out as previously described at 19°C (Diaz and Viney 2014). Briefly on Day 1, a synchronized L1 was introduced to a plate with an excess of food (Hope 1999), and then transferred to a fresh plate every other day during its reproductive life. Egg-containing plates were incubated for an extra 48 h, and then the viable larvae counted. The number of viable offspring was then used to describe a worm's lifetime fecundity. We compared the early (days 1 and 3) and late (days 5 and 7) reproduction. To measure survival each adult worm was monitored every other day until it was recorded as dead. Lifetime fecundity and survival were measured for five individuals *per* isogenic line (with each worm defined as a replicate within a line) of each selection regime and of the control population.

### Data analysis

We used generalized linear mixed-effects models (GLMM) to investigate changes in the dauer larval formation of isogenic lines arising from the four selection regimes, compared with lines from the control population. For the model, we used the logit function with a binomial distribution to describe the proportion of dauer larvae (*p*) and non-dauer larvae (*q)* among the selection Regime lines and Food concentration treatments at the phenotyping phase, and sample size (*n*) per dauer larvae assay plate. For model construction we started with the simplest null model that included only the overall mean of dauer larvae formation across the data, and then we added explanatory variables and their interactions sequentially.

We used GLMM (with Poisson error distribution) to analyse the count data of the lifetime fecundity and the time of reproduction of the lines using a log-link function. For the survival data, we used a mixed-effect Cox model (MECM). In all these models, we analysed variation in these traits in response to selection Regime and compared them to the control population.

For each analysis, a series of candidate models were constructed to evaluate the effect of each explanatory variable and their interactions. When it was applicable, a random effect was included in each model to account for the repeated observations of each isogenic line. GLMM models were compared using the Akaike Information Criterion (AIC) and MECM models using log-likelihood ratio test. Model results are presented in the Appendix. Analyses were performed using R software (R core Team 2013). Unless otherwise stated dauer larva formation is reported as the number of dauer larvae as a proportion of all larvae (i.e., the number of dauer larvae and of non-dauer larvae) shown as the mean ± standard error of the mean (SEM) of three replicates of each isogenic line. For both the assays of dauer larva formation and of lifetime fecundity, the data presented for each isogenic line are the mean across replicates.

## Results

Five generations of selection in four different selection regimes successfully selected for altered dauer larva formation phenotypes. At generation five, the proportion of worms that were selected as dauer larvae differed significantly between the one and two cue selective regimes (*t*-value = 2.73, *P*-value < 0.001; Appendix Table3), with twice as many being selected in the latter (3.51 ± 2.13% and 7.24 ± 1.20% in one ((i) and (ii)) or two ((iii) and (iv)) cue regimes, respectively, at generation 5; Appendix Fig.4, Table3).

**Table 1:** Changes in dauer larva formation (mean ± SEM) in response to the two food environments among the four selection regimes compared with the control, unselected population (GLMM analysis in Appendix Table4)

	Control	Selection regime			
Food treatments		(i) 2% food	(ii) 5% food	(iii) 2% food + ascr#2	(iv) 5% food + ascr#2
2% food	0.19 ± 0.04	0.14 ± 0.03 n.s.	0.56 ± 0.03**	0.78 ± 0.08***	0.89 ± 0.07***
5% food	0.10 ± 0.01	0.02 ± 0.01 n.s.	0.14 ± 0.01 n.s.	0.51 ± 0.12 n.s.	0.42 ± 0.15***

n.s. = not significant, ^**^P < 0.01, ^***^P < 0.001.

**Figure 2:**
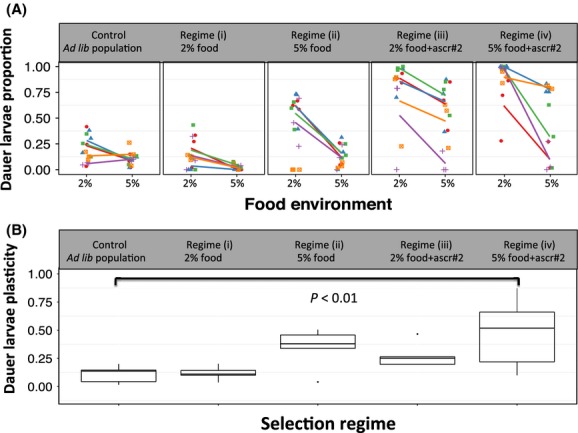
(A) The proportion of dauer larvae formed by lines selected in regimes (i)–(iv), and of the control, unselected population, tested in two food (2% and 5% w/v) environments. Each line and colour represents the mean dauer formation of each inbred line, with the data for each repeat shown by symbols, where each symbol shape is one inbred line. (B) Variation in the plasticity for dauer larva formation calculated as the absolute difference in dauer larvae formation between the 2% and 5% food environments. The boxplot shows the median (horizontal line), upper and lower quartiles (box), the data range (whiskers), and outliers (circles). *P*-values show changes in the plasticity of dauer larva formation compared to the control population (Appendix Table4).

After five generations of selection we phenotyped lines from the selection and control regimes in two different food environments. The control, unselected, population produced a very low proportion of dauer larvae in either food environment (0.19 ± 0.04 and 0.10 ± 0.01 at 2% and 5% food, respectively, *z*-value = −1.06, *P*-value = 0.29 Fig.2A, Table1; Appendix Table4). We found no differences in dauer larva formation in response to a change in the food concentration, thus a very low plasticity of dauer larva formation among these lines (Fig.2B; Appendix Table4).

In the lines resulting from the four selection regimes, there was a difference in the proportion of dauer larvae that developed, compared with the control population, when tested in both food concentration conditions (best model: Regime × Food, Fig.2, Table2). This shows that the dauer larva formation response of the lines evolved differently among the four selection regimes.

**Table 2:** Generalized linear mixed-effect model (GLMM) selection describing the dauer larvae formation response in relation to Regime and Food variables showing the explanatory variables in the model, the number of parameters (*K*) and the Akaike Information Criterion (AIC) value. The best model is shown in bold. Note that *K* includes the random term of the replicate within line and within food

Model	*K*	AIC
Null model	2	2105.42
REGIME	6	1833.41
FOOD	3	2101.30
REGIME + FOOD	7	1817.73
**REGIME × FOOD**	**11**	**1754.66**

Selection regime (i) (i.e., 2% food, no ascr#2) resulted in lines that did not differ from the control lines (0.14 ± 0.03 and 0.02 ± 0.01 in 2% and 5% food concentration, respectively, *z*-value = −0.63 and −1.90, *P*-value = 0.53 and 0.06, respectively, Fig.2A, Tables2; Appendix Table4). This suggests that the plasticity of dauer larva formation did not evolve (Fig.2B). Selection in regime (ii) (i.e., 5% food, no ascr#2) resulted in an increase in dauer larva formation in the low food concentration environment (0.56 ± 0.03 proportion; *z*-value = 2.59, *P* < 0.01; Tables2; Appendix Table4), but there was no change in the high food concentration environment (0.14 ± 0.01 proportion; *z*-value = −1.62, *P* = 0.11). Notwithstanding this change, the plasticity of these lines remained similar to the control lines (Fig.2B).

Selection in regime (iii) (i.e., 2% food + ascr#2) resulted in lines with increased dauer larvae formation in the low food concentration environment (0.78 ± 0.08 proportion; *z*-value = 6.10, *P* < 0.001; Fig.2A, Table1; Appendix Table4), but no change in dauer larvae formation in the high food concentration environment, compared with the control lines (0.51 ± 0.12 proportion; *z*-value = −1.48, *P* = 0.14; Table1; Appendix Table4). Thus, similar to selection regime (ii), despite the increase in dauer larvae formation in the low food concentration environment there was no change in the plasticity of these lines (Fig.2B).

Selection in regime (iv) resulted in an increase in dauer larvae formation in both the low and high food concentration environments (0.89 ± 0.07 and 0.42 ± 0.15 proportion, respectively, *z*-value = 7.95 and −3.52, both: *P* < 0.001; Fig.2A, Table1; Appendix Table4). These changes resulted in an increase in the plasticity of dauer larva formation (Fig.2B; Appendix Table5).

**Figure 3:**
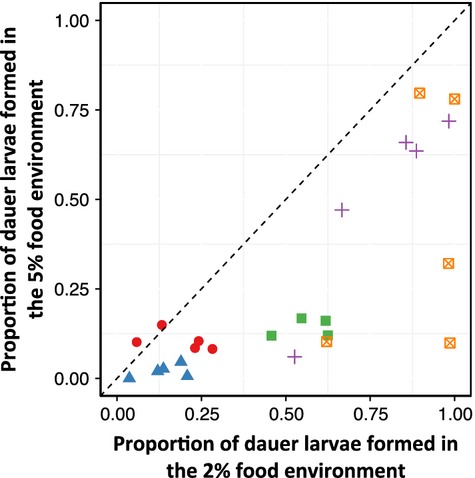
The correlation of dauer larva formation by selected and control lines tested in 2% and 5% w/v food environments, where each dot is the mean value for each inbred line from across three replicates, and where the symbols and colours differentiate the four different selection regimes (blue = 2% food, green = 5% food, violet = 2% food & ascr#2, and orange = 5% food & ascr#2) and the unselected, control population (red).

Across the four selection regimes and the control population there was a positive correlation in the lines’ dauer larva formation across the two food concentration treatments (Spearman's correlation = 0.78, *P* < 0.001; Fig.3). Thus, despite the differences in dauer larvae formation among the four selection regimes (Fig.2A), it suggests that the evolved dauer larva formation responses to each food environment are not independent.

There were no differences in the lifetime fecundity of worms (i) between lines from the selection regimes and the control population or (ii) among lines from the four selection regimes (Appendix Tables6, and Fig.5); on average, worms produced 197.84 ± 6.12 progeny. However, lines differed in the distribution of the proportion of their lifetime fecundity among different days of reproduction (Appendix Tables8, Fig.6). Overall, the selected lines reproduced earlier, compared to the control lines (Appendix Fig.6); worms from the control unselected population produced about a third of their offspring between days 1 and 3, whereas worms from the selection regimes produced *ca*. 80% of theirs in the same period. There were no differences in the survival of worms from the selected lines compared to the control population (*χ*^2^ = 5.50, df = 4, *P* > 0.05; Appendix Table10); on average, worms lifespan was 19.07 ± 0.30 days (Appendix Fig.7).

## Discussion

Our results show that in *C. elegans* the trait of dauer larva formation can be selected. Selection in most regimes resulted in an increase in the average dauer larva formation across the two food concentration environments, suggesting that dauer larva development can rapidly evolve given sufficient genetic variation and a selection pressure. Moreover, selection regime (iv) (high food + ascr#2) also changed the plasticity of dauer larva formation, suggesting that this two cue combination is a stronger plasticity selecting environment.

We found that one of the two cue selection regimes resulted in significantly greater dauer larva formation phenotypes when lines were tested in a one cue food environment, compared with single cue selection regimes. Comparing the response to selection in regime (ii) with that in regime (iv) shows that the addition of ascr#2 results in the evolution of trait plasticity, but to a change in the concentration of food in the environment in the absence of ascaroside. In the *C. elegans* dauer larva formation pathway environmental conditions are first sensed, this environmental information is then integrated, and this is then used to make a decision to execute the dauer or the nondauer developmental program (Riddle and Albert 1997). These results suggest that the molecular processes by which cues are sensed and the integration of this information are environmentally dependent, such that an evolved change in one environment has a consequent effect on the other environment. Moreover, the positive correlation of dauer larvae formation between the two food environments may suggest that the responses to different food environments are not completely independent. The mechanism underlying this is beyond the scope of the work that we present, but it could, for example, be due to the effect of allele(s) whose effect is to increase dauer larva in both food environments.

We found that the greatest response to selection that we observed was in the high food concentration + ascaroside environment, regime (iv). Typically, a low food concentration is a stronger dauer larva formation cue than a high food concentration cue (Diaz et al. 2014). Therefore, selection regime (iv) may be a combination of weakly (high food concentration) and strongly (ascaroside) dauer larva-inducing cues. This suggests that the strong response to selection in regime (iv) is not a simple synergistic effect between two strong cues (which is our regime (iii)). These results suggest that some aspect of the genetic network by which the dauer larva formation plasticity is controlled has been specifically selected in this two cue environment, resulting in the change to this plasticity.

Beyond dauer larva formation phenotypes, the lines also evolved to alter their schedule of reproduction so that they reproduced earlier. These effects appear to be consequent, correlated effects of the evolution of dauer larva formation. The schedule of the actual selection regime is unlikely to be the cause of this change, because the control unselected lines were maintained on the same schedule. Ascarosides #2 and #3 have been shown to slightly promote fecundity in *C. elegans* (Ludewig et al. 2013). These and our results together point to these ascarosides having effects on reproduction in *C. elegans*.

*Caenorhabditis elegans* produces almost 150 different ascaroside and related molecules (von Reuss et al. 2012). These molecules have multiple effects on *C. elegans* including inducing dauer larva formation, dauer larva dispersal, and male attraction (Diaz et al. 2014). Ascaroside #2 is the most potent dauer larva-inducing ascaroside, but it acts synergistically with at least five others (Butcher et al. 2007; Srinivasan et al. 2008; Butcher et al. 2008; Kaplan et al. 2011; Diaz et al. 2014). Therefore, while we considered one and two cue selective environments with respect to dauer larva formation, the actual context in which *C. elegans* evolves its plasticity of dauer larva formation is a very substantially richer and more complex cue environment.

Dauer larvae play a key role in the persistence of *C. elegans* in ephemeral environments. The hitherto canonical view of *C. elegans* dauer larva formation is that they are formed in a low food, high conspecific population density (measured by the worm-derived ascaroside environment) environment (Viney et al. 2003). But, more recent work has shown that some worm isolates have an opposite plasticity, so that dauer larva formation can be favored in comparatively richer food environments (Diaz et al. 2014). The results of our selection experiments suggest that dauer larva formation phenotypes have the potential to be very malleable. One can therefore envisage that other selection regimes could produce the full range of dauer larva, arrested development phenotypes seen in wild-derived genotypes (Diaz et al. 2014).

## Figures and Tables

**Figure A1:**
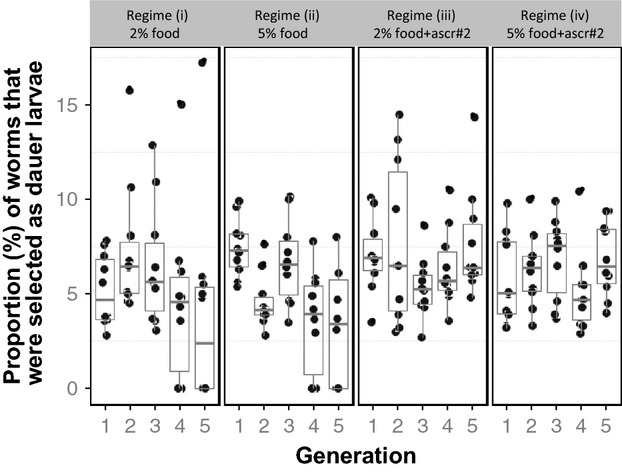
The proportion (%) of worms that were selected as dauer larvae at each generation of selection across the four selection regimes. Each dot represents the proportion of larvae that survived on each replicate plate at each generation, and the boxplot shows the median (horizontal line), upper and lower quartiles (box), and the data range (whiskers).

**Figure A2:**
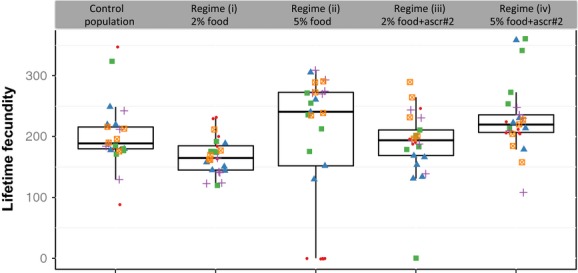
The lifetime fecundity of lines selected in regimes (i)–(iv) and of the control, unselected population. Each dot is the lifetime fecundity of one adult hermaphrodite, with the different symbols and colours identifying worms from one isogenic line. Boxplot represent the median (horizontal line), upper and lower quartiles (box), data range (whiskers), and outliers.

**Figure A3:**
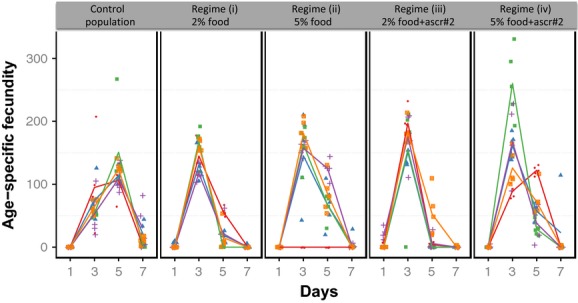
The age-specific fecundity of lines selected in regimes (i)–(iv) and of the control, unselected population. Each dot is the age-specific fecundity of an individual adult hermaphrodite during a time interval (thus Day 1 is a 48-h period starting at, and including, Day 1), with the different symbols and colours identifying worms from one isogenic line, and the lines showing the mean value across these individual worms.

**Figure A4:**
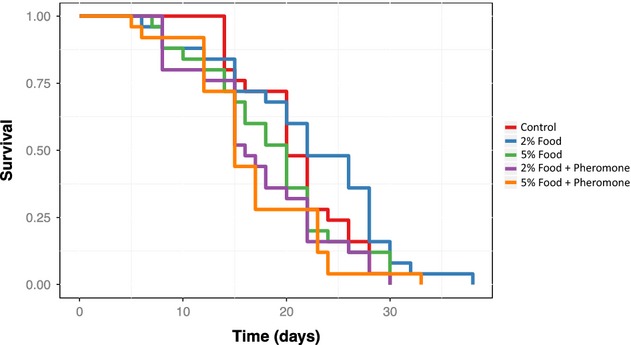
The survival of lines selected in regimes (i)–(iv) and of the control, unselected population, with each color representing each selection or control regime.

## Appendix

**Table A1:** (A) Generalized linear model (GLM) selection describing the proportion of worms that were selected as dauer larvae in response to generation, the presence or absence of ascaroside, and food environments. Models were fitted using a normal error distribution, showing the explanatory variables in the model, the number of parameters (*K*) and the Akaike Information Criterion (AIC) value. The best model is shown in bold. (B) GLM describing the best model. The intercept represents the proportion of worms that were selected as dauer larvae in the single cue environments (i.e., an absence of ascaroside) at generation 1. There were 200 observations, grouped within 10 plates per regime per generation

(A)		
Model	*K*	AIC
Null model	1	1325.86
GENERATION	3	1010.09
ASCAROSIDE	3	1007.58
FOOD	3	1014.35
GENERATION + ASCAROSIDE	4	1003.56
**GENERATION × ASCAROSIDE**	**5**	**998.05**
GENERATION × ASCAROSIDE + FOOD	6	998.36
GENERATION × ASCAROSIDE × FOOD	9	1030.12

(B)				
Variable	Estimate	Std. Error	*t* value	*P*-value
(Intercept)	7.55	0.67	11.14	<0.001
GENERATION	−0.72	0.20	−3.70	<0.001
ASCAROSIDE	−1.15	0.95	−1.20	0.23
GENERATION × ASCAROSIDE	0.79	0.28	2.73	<0.001

**Table A2:** GLMM with a binomial error distribution (logit function) describing changes in dauer larvae formation among the four selection regimes and the control, unselected population. The lines were then tested in 2% or 5% food treatments. The intercept represents dauer larva formation of the control population in a 2% food treatment. The model contains fixed and random effects (explanatory variables and replicate effects, respectively). Model syntax: (dauer, no_dauers) ∽ Regime × Food + (1 | line:food). There were 150 observations, grouped within three replicates within five lines, for the four selection regimes plus the control, unselected population, tested in two food environments

Fixed effects	Estimate	Std. Error	*z* value	*P*-value
(Intercept)	−1.63	0.37	−4.37	<0.001
2% Food Regime	−0.33	0.52	−0.63	0.53
5% Food Regime	1.35	0.52	2.59	<0.01
2% Food + ascr#2 Regime	3.20	0.52	6.10	<0.001
5% Food + ascr#2 Regime	4.24	0.53	7.95	<0.001
5% Food treatment	−0.56	0.53	−1.06	0.29
2% Food Regime: 5% Food treatment	−1.48	0.78	−1.90	0.06
5% Food Regime: 5% Food treatment	−1.20	0.74	−1.62	0.11
2% Food + ascr#2 Regime: 5% Food treatment	−1.09	0.74	−1.48	0.14
5% Food + ascr#2 Regime: 5% Food treatment	−2.63	0.75	−3.52	<0.001

Random effects	Variance	Std. Dev.
Replicate within line within food (Intercept)	0.63	0.79

**Table A3:** Generalized linear model selection describing the plasticity of dauer larvae formation in relation to REGIME, showing the explanatory variables in the model, the number of parameters (*K*), and the Akaike Information Criterion (AIC) value. The best model is shown in bold. Note, the *K* number includes the residual variance

Model	*K*	AIC
Null model	2	−0.494
**REGIME**	**6**	−**9.001**

**Table A4:** GLMM selection describing the lifetime fecundity in relation to REGIME, showing the explanatory variables in the model, the number of parameters (*K*), and the Akaike Information Criterion (AIC) value. The best model is shown in bold. Note, the *K* number includes the random term of the replicate within line

Model	*K*	AIC
**Null model**	**2**	**2222.03**
REGIME	6	2226.94

**Table A5:** GLMM with a Poisson error distribution (logit function) describing changes in lifetime fecundity among the selection regimes and the control population. The intercept is the overall mean across the data (Table A4). Model syntax: fecundity.sum ∽ 1 + (1 | line). There were 125 observations grouped in five replicates within five lines, for the four selection regimes plus the control, unselected population

Fixed effects	Estimate	Std. Error	*z* value	*P*-value
(Intercept)	5.09	0.22	22.93	<0.001

Random effects	Variance	Std. Dev.
Replicate within line (Intercept)	1.22	1.10

**Table A6:** GLMM selection comparing early (days 1 and 3) and late (days 5 and 7) reproduction of worms in REGIME to regime and day variables, showing the explanatory variables in the model, the number of parameters (*K*), and the Akaike Information Criterion (*AIC*) value. The best model is shown in bold. Note, the *K* number includes the random term of individual within replicate within line within regime

Model	*K*	AIC
Null model	2	13917.73
DAY	3	10326.96
REGIME	6	13915.52
REGIME + DAY	7	10324.75
**REGIME × DAY**	**11**	**5232.83**

**Table A7:** GLMM with a Poisson error distribution (logit function) describing changes in the time of reproduction among selection regimes and the control population. The intercept represents the number of progeny produced early (days 1 and 3) by worms in the control, unselected population. The model contains fixed and random effects (explanatory variables and replicate effects, respectively). Model syntax: age.fecundity ∽ Regime × Day + (1 | line:individual). It contains 500 observations grouped within five individual observations within five lines, for the four selection regimes plus the control, unselected population

Fixed effects	Estimate	Std. Error	*z* value	*P*-value
(Intercept)	4.18	0.17	25.10	<0.001
2% Food Regime	0.78	0.24	3.32	<0.001
5% Food Regime	0.07	0.24	0.29	0.77
2% Food + ascr#2 Regime	0.86	0.24	3.66	<0.001
5% Food + ascr#2 Regime	0.87	0.24	3.68	<0.001
Day 5–7	0.68	0.03	22.85	<0.001
2% Food Regime: Day 5–7	−2.53	0.05	−46.91	<0.001
5% Food Regime: Day 5–7	−1.29	0.04	−30.73	<0.001
2% Food + ascr#2 Regime: Day 5–7	−3.32	0.07	−50.76	<0.001
5% Food + ascr#2 Regime: Day 5–7	−1.54	0.04	−37.01	<0.001

Random effects	Variance	Std. Dev.
Individual within replicate within line	0.68	0.82

**Table A8:** Mixed-effect Cox model (MECM) selection describing the survival in relation to REGIME, showing the explanatory variables in the model, the number of parameters (*K*), and log-likelihood (logLik) value. The best model is shown in bold. Note, the *K* number includes the random term of the replicate within line

Model	*K*	logLik
**Null model**	**3**	−**472.57**
REGIME	7	−469.82

## References

[b1] Butcher RA, Fujita M, Schroeder FC, Clardy J (2007). Small-molecule pheromones that control dauer development in *Caenorhabditis elegans*. Nat. Chem. Biol.

[b2] Butcher RA, Ragains JR, Kim E, Clardy J (2008). A potent dauer pheromone component in *Caenorhabditis elegans* that acts synergistically with other components. Proc. Natl Acad. Sci. USA.

[b3] Chen S, Glazer I (2004). Effect of rapid and gradual increase of osmotic stress on survival of entomopathogenic nematodes. Phytoparasitica.

[b4] Danks HV (1987). Insect dormancy: an ecological perspective.

[b5] Denlinger DL (2002). Regulation of diapause. Annu. Rev. Entomol.

[b6] Diaz SA, Viney ME (2014). Genotypic-specific variance in *Caenorhabditis elegans* lifetime fecundity. Ecol. Evol.

[b7] Diaz A, Lloyd-Jones G, Spinner W, Wharam B, Viney ME (2014). Diverse and potentially dishonest signalling with ascarosides in the model nematode *C. elegans*. BMC Evol. Biol.

[b8] Félix M-A, Braendle C (2010). The natural history of *Caenorhabditis elegans*. Curr. Biol.

[b9] Golden JW, Riddle DL (1984). The *Caenorhabditis elegans* dauer larva: developmental effects of pheromone, food and temperature. Dev. Biol.

[b10] Hope IA (1999). C. elegans: a practical approach.

[b11] Hu PJ (2007). 10.1895/wormbook.1.144.1.

[b12] Kaplan F, Srinivasan J, Mahanti P, Ajredini R, Durak O, Nimalendran R (2011). Ascaroside expression in *Caenorhabditis elegans* is strongly dependent on diet and developmental stage. PLoS One.

[b13] Ludewig AH, Schroeder FC (2014). 10.1895/wormbook.1.155.1.

[b14] Ludewig AH, Izrayelit Y, Park D, Malik RU, Zimmermann A, Mahanti P (2013). Pheromone sensing regulates *Caenorhabditis elegans* lifespan and stress resistance via the deacetylase SIR-2.1. Proc. Natl Acad. Sci. USA.

[b15] Mayer MG, Sommer RJ (2011). Natural variation in *Pristionchus pacificus* dauer formation reveals cross-preference rather than self-preference of nematode dauer pheromones. Proc. R. Soc. Lond. B.

[b16] Petersen C, Dirksen P, Prahl S, Strathmann EA, Schulenburg H (2014). The prevalence of *Caenorhabditis elegans* across 1.5 years in selected North German locations: the importance of substrate type, abiotic parameters and *Caenorhabditis* competitors. BMC Ecol.

[b17] von Reuss SH, Bose N, Srinivasan J, Yim JJ, Judkins JC, Sternberg PW (2012). Comparative metabolomics reveals biogenesis of ascarosides, a modular library of small-molecule signals in *C. elegans*. J. Am. Chem. Soc.

[b118] R Core Team (2013). R: A Language and environmental for statistical computing. http://www.R-project.org/.

[b18] Riddle DL, Riddle DA, Blumenthal T, Meyer BJ, Priess JR, Albert PS (1997). Genetic and environmental regulation of dauer larva development. C. elegans II.

[b19] Riddle DL, Swanson MM, Albert PS (1981). Interacting genes in nematode dauer larva formation. Nature.

[b20] Srinivasan J, Kaplan F, Ajredini R, Zachariah C, Alborn HT, Teal PEA (2008). A blend of small molecules regulates both mating and development in *Caenorhabditis elegans*. Nature.

[b21] Teotonio H, Carvalho S, Manoel D, Roque M, Chelo IM (2012). Evolution of out crossing in experimental populations of *Caenorhabditis elegans*. PLoS One.

[b22] Viney ME, Diaz A (2012). Phenotypic plasticity in nematodes: evolutionary and ecological significance. Worm.

[b23] Viney ME, Gardner MP, Jackson JA (2003). Variation in *Caenorhabditis elegans* dauer larva formation. Dev. Growth Differ.

[b24] West-Eberhard MJ (2003). Developmental plasticity and evolution.

